# A Greater Improvement of Intrahepatic Fat Contents after 6 Months of Lifestyle Intervention Is Related to a Better Oxidative Stress and Inflammatory Status in Non-Alcoholic Fatty Liver Disease

**DOI:** 10.3390/antiox11071266

**Published:** 2022-06-27

**Authors:** Margalida Monserrat-Mesquida, Magdalena Quetglas-Llabrés, Cristina Bouzas, Sofía Montemayor, Catalina M. Mascaró, Miguel Casares, Isabel Llompart, José M. Gámez, Silvia Tejada, J. Alfredo Martínez, Josep A. Tur, Antoni Sureda

**Affiliations:** 1 Research Group in Community Nutrition and Oxidative Stress, University of the Balearic Islands—IUNICS, 07122 Palma de Mallorca, Spain; margalida.monserrat@uib.es (M.M.-M.); m.quetglas@uib.es (M.Q.-L.); cristinabouzas@uib.es (C.B.); sofiamf16@gmail.com (S.M.); c.mascaro@uib.es (C.M.M.); isabel.llompart@ssib.es (I.L.); jmgamez@hsll.es (J.M.G.); silvia.tejada@uib.es (S.T.); antoni.sureda@uib.es (A.S.); 2 Health Research Institute of Balearic Islands (IdISBa), 07120 Palma de Mallorca, Spain; 3 CIBER Fisiopatología de la Obesidad y Nutrición (CIBEROBN), Instituto de Salud Carlos III (ISCIII), 28029 Madrid, Spain; 4 Radiodiagnosis Service, Red Asistencial Juaneda, 07011 Palma de Mallorca, Spain; casaresmiguel@gmail.com; 5 Clinical Analysis Service, University Hospital Son Espases, 07198 Palma de Mallorca, Spain; 6 Cardiology Service, University Hospital Son Llàtzer, 07010 Palma de Mallorca, Spain; 7 Laboratory of Neurophysiology, Department of Biology, University of the Balearic Islands, 07122 Palma de Mallorca, Spain; 8 Cardiometabolics Precision Nutrition Program, Instituto Madrileño de Estudios Avanzados de la Alimentación (IMDEA Food-CEI UAM-CSIC), 28049 Madrid, Spain; jalfredo.martinez@imdea.org

**Keywords:** NAFLD, IFC, aerobic capacity, oxidative stress, inflammation, biomarkers

## Abstract

Non-alcoholic fatty liver disease (NAFLD) is a disorder characterized by the excessive accumulation of lipids in the liver parenchyma. To date, there is no effective pharmacological treatment against NAFLD. Objective: To assess the relationship between the improvement of the intrahepatic fat content (IFC) in patients with NAFLD and metabolic syndrome and biomarkers of oxidative stress and inflammation after 6 months of lifestyle intervention. Patients diagnosed with NAFLD (*n* = 60 adults; 40–60 years old) residing in the Balearic Islands, Spain, were distributed in tertiles attending the improvement of IFC calculated by magnetic resonance imaging (MRI). Anthropometrics, blood pressure, maximal oxygen uptake, and pro/antioxidant and inflammatory biomarkers were determined in plasma before and after the lifestyle intervention. The improvement in IFC levels was higher in tertile 3 with respect to tertiles 2 and 1. The greatest improvement in IFC is related to cardiorespiratory fitness and adherence to the Mediterranean diet (ADM). Higher reductions in weight, body mass index (BMI), and alanine aminotransferase (ALT) were observed in tertile 3 with respect to tertile 1 after 6 months of intervention. The improvement in catalase, irisin, and cytokeratin 18 plasma levels were higher in tertile 3, whereas no differences were observed in superoxide dismutase activity. Malondialdehyde and protein carbonyl levels, as biomarkers of oxidative damage, remained unchanged in all groups. The present data show that the reduction of IFC is associated with an improvement in pro/antioxidant and pro-inflammatory status and a better cardiorespiratory fitness in NAFLD patients.

## 1. Introduction

Non-alcoholic fatty liver disease (NAFLD) is an epidemic liver disorder characterized by excessive accumulation of lipids in the liver parenchyma [1]. This disease is the most common chronic liver disease, and its prevalence has been progressively increased in recent years, like the worldwide increase in diabetes and metabolic syndrome [2]. Nowadays, NAFLD affects about 20–30% of the global population, but it has 90% prevalence among obese individuals [3,4]. If the pathological disorder is not appropriately treated, it can progress from NAFLD to the more advanced stage of non-alcoholic steatohepatitis (NASH), which can, ultimately, lead to cirrhosis and liver cancer [5]. Moreover, NAFLD is related to metabolic disorders with extrahepatic manifestations, such as cardiovascular disease, chronic kidney disease, sleep apnea, obesity, insulin resistance, and diabetes [3,6]. Although the factors responsible for the onset and progression of hepatic steatosis are not well-elucidated, lifestyle, genetics, immunity, and the gut microbiota may be involved [7]. Lifestyle risk factors such as smoking, unhealthy diet, and reduced physical activity significantly increase the risk of hepatic steatosis [8,9].

Numerous evidence suggested that oxidative stress induced by increased production of reactive species (ROS) is involved in the pathogenesis of many diseases, including metabolic disorders such as insulin resistance or diabetes [10]. Oxidative stress (OS) and inflammation induced by the excess of ROS are well-recognized mechanisms that can lead to tissue injury and hepatic cell death [11]. In this sense, NAFLD has been described as a pro-oxidative and pro-inflammatory disease [12]. In NAFLD patients, the accumulation of fatty acids in the liver induces an increase in β-oxidation, which, in turn, causes a ROS overproduction in the respiratory chain [11]. This increase in ROS production can exceed the detoxifying capacity of the antioxidant system, leading to the appearance of lipid peroxidation and oxidative damage [13]. All this leads to impaired mitochondrial and peroxisomal oxidation of fatty acids that can progress and result in reduced hepatic ATP synthesis and caspases-induced apoptosis [14]. ROS also induces an increase of the transforming grow factor β (TGF-β), which activates collagen production by stellate cells, increasing hepatic fibrosis [15]. In addition, previous studies have also demonstrated that NAFLD is related to up-regulation of pro-inflammatory mediators [16,17]. In fact, the excessive accumulation of triglycerides and lipotoxic intermediates causes changes in hepatocyte function and induces the release of pro-inflammatory cytokines such as tumor necrosis factor-α (TNF-α) or interleukin-6 (IL-6), which, together with circulating non-esterified fatty acids, disturb hepatic insulin signaling [18,19].

Consistent evidence support common pathophysiologic mechanisms between MetS and NAFLD, which usually involve visceral obesity and insulin resistance [20]. Currently, there is no drug therapy that can be formulated for treating NAFLD. A combination of a healthy diet, such as Mediterranean diet [21], and increased physical activity [22] remains the mainstay in the management of NAFLD. Thus, lifestyle intervention can be effective for treating NAFLD patients, including a healthy diet and increased physical activity [23]. In this sense, it has been shown that weight reductions could decrease cardiovascular diseases and the risk of diabetes as well as can reverse liver diseases, leading to the improvement of fibrosis [24,25,26,27]. Moreover, it has been shown that the intrahepatic lipid content in people with NAFLD can be reduced by regular exercise training [27]. Regular exercise can improve NAFLD by diverse mechanisms such as decreasing intrahepatic fat content, producing hepato-protective autophagy, rising β-oxidation of fatty acids, overexpressing peroxisome proliferator-activated receptor-γ (PPAR-γ), rising insulin sensitivity, and attenuating hepatocyte apoptosis [28].

Considering the relationship between excessive fat accumulation in tissues and the proinflammatory and pro-oxidative state, the aim of this study was to assess whether a greater improvement in intrahepatic fat contents (IFC) in patients with NAFLD undergoing a lifestyle intervention also leads to an improvement in the proinflammatory and oxidative state.

## 2. Materials and Methods

### 2.1. Design and Participants

The current study was included within the frame of the FLIPAN (Prevention and Reversion of NAFLD in Obese Patients with Metabolic Syndrome by Mediterranean Diet and Physical Activity) prospective and randomized control trial (ClinicalTrials.gov Identifier: NCT04442620). It involves 60 participants residing in the Balearic Islands (Spain), aged 40–60 years, with a diagnosis of NAFLD by magnetic resonance imaging (MRI), a BMI of 27–40 kg/m^2^, and showing at least three of the metabolic syndrome (MetS) criteria as described by the International Diabetes Federation (IDF) consensus [29]. The participants were selected considering the inclusion criteria described elsewhere [12]. Exclusion criteria were previous cardiovascular disease, congestive heart failure, liver diseases (other than NAFLD), cancer or a history of malignancy in the previous 5 years, previous bariatric surgery, acute febrile illnesses, urinary tract infections, post-renal hematuria, hemochromatosis, protein overload, non-medicated depression or anxiety, alcohol and drug abuse, pregnancy, primary endocrinological diseases (other than hypothyroidism and type 2 diabetes mellitus), concomitant therapy with steroids, intense physical exercise, or being unable to provide informed consent.

All procedures and the study protocol were designed according to the ethical standards of the Declaration of Helsinki and were approved by the Ethics Committee of the Balearic Islands (ref. IB 2251/14 PI). The participants were informed of the purposes and the potential risks of the study and signed the informed consent to participate.

After inclusion, participants were randomly allocated to one of the following three groups:

1. Conventional diet (CD) group: These participants followed the recommendations of American Association for the Study of Liver Disease (AASLD) [30], with energy restrictions to loss of at least 3–5% of the body weight to improve steatosis and 7–10% to improve most of the histopathological features of NASH, following the general guidelines of the U.S. Department of Health and Human Services and U.S. Department of Agriculture (20–35% fat, 10–35% protein, 45–65% carbohydrate) [31].
2. Mediterranean diet high meal frequency (MD-HMF) group: This group was instructed to follow a Mediterranean diet characterized by a distribution of macronutrients of 40–45% carbohydrates (50–70% of carbohydrates should be low glycemic and rich in fiber), 30–35% fat, and 25% protein. This dietary pattern was previously observed to decrease fat mass and overall weight and improve the oxidative status in subjects with metabolic syndrome [32,33]. Total daily caloric intake was distributed over seven meals, with the highest calorie meals eaten early during the morning.
3. Mediterranean diet physical activity (MD-PA) group: This group consumed an energy-restricted Mediterranean diet with a meal frequency of four to five meals per day, including snacks. Total calorie intake for this group came from 35–40% from fat (8–10% of saturated fatty acids, >20% of monounsaturated fatty acids, >10% of polyunsaturated fatty acids, and <300 mg/day of cholesterol), about 20% from proteins, and 40–45% or more from carbohydrates (mainly with low glycemic index). Sodium chloride should not reach 6 g/day (2.4 g of sodium), and dietary fiber should be no less than 30–35 g/day [34].

The CD and MD-HMF groups were instructed to perform at least 10,000 steps a day [19], and the MD-PA group was instructed to undergo 35 min interval training session three times a week as previously described [35]. The three nutritional interventions were characterized by an energy reduction of 25–30% of baseline calories intake and increase energy expenditure by 400 kcal/70 kg (5.7 kcal per kg of body weight). The adherence to the Mediterranean diet (ADM) was assessed using a validated 17-item questionnaire at the beginning and after 6 months of intervention [36].

### 2.2. Anthropometric Characterization

Weight (kg) was determined with a calibrated scale with the patients barefoot and light clothing, therefore subtracting 0.6 kg for their clothing. Height was measured with a mobile anthropometer (Seca 214, SECA Deutschland, Hamburg, Germany) to the nearest millimeter, keeping the patient’s head in the Frankfort horizontal plane position. Body mass index (BMI) was calculated as kg/m^2^. Intrahepatic fat content (IFC) was determined using a 1.5-T magnetic resonance imaging (MRI) (Signa Explorer 1.5T, General Electric Healthcare, Chicago, IL, USA) equipped with a 12-channel phased-array coil [37]. Blood pressure was determined in triplicate in the sitting position using a validated semi-automatic oscillometer (Omron HEM, 750CP, Hoofddrop, The Netherlands). The maximal oxygen uptake (VO_2_ max) was measured with Chester step test (CST) [38].

### 2.3. Blood Collection and Analysis

Venous blood samples were collected from the antecubital vein with vacutainers containing the anticoagulant ethylene diamine tetra acetic acid (EDTA), after 12-hour overnight fasting. Plasma was isolated by centrifuging the fresh blood at 1700× *g* for15 min at 4 °C. Biochemical parameters were determined using standardized clinical procedures. The hematological parameters and cell counts were analyzed in whole blood (automatic flow cytometer analyzer Technion H2, Bayer, VCS system, Frankfurt, Germany).

### 2.4. Enzymatic Determinations

Plasma was used to measure the activities of the antioxidant enzymes catalase (CAT) and superoxide dismutase (SOD) using Shimadzu UV-2100 spectrophotometer (Shimadzu Corporation, Kyoto, Japan) at 37 °C. Specifically, the activity of CAT was determined monitoring the decomposition of H_2_O_2_ following the method of Aebi [39]. SOD activity was determined by an adapted method of Flohe and Otting based on the inhibition of the reduction of cytochrome C by superoxide anion generated by the xanthine oxidase/hypoxanthine system [40].

### 2.5. Malondialdehyde Assay

Malondialdehyde (MDA), a marker of lipid peroxidation, was analyzed in plasma using a colorimetric assay kit (Merck Life Science S.L.U., Madrid, Spain). Briefly, samples and MDA standard were positioned in tubes holding *n*-methyl-2-phenylindole in acetonitrile: methanol (3:1). Then, HCL (12N) was added, and tubes were incubated at 45 °C for 1 h. Finally, the absorbance was determined at 586 nm.

### 2.6. Protein Carbonyl Determination

Protein carbonyl derivatives were determined by an OxiSelectTM Protein Carbonyl Immunoblot Kit (CELL BIOLABS^®^, San Jose, CA, USA) following the supplied guidelines for use. The total protein levels in the samples were determined by the Bradford method [41] using a commercial reagent (Merck Life Science S.L.U., Madrid, Spain). Briefly, following the dot blot method (Bio-Rad, Hercules, CA, USA), 10 µg of protein was transferred into a nitrocellulose membrane, which was incubated with 2,4-dinitrophenylhydrazine (DNPH). Then, the membrane was incubated with the primary antibody specific to DNPH (1:1000). This step was followed by incubation with goat antirabbit IgG (1:1000). After that, immunoblot development was carried out using an enhanced chemiluminescence kit (Immun-Star Western C Kit reagent, Bio-Rad Laboratories, Hercules, CA, USA). Finally, the protein carbonyl bands were quantified with the image analysis program, Quantity One (Bio-Rad Laboratories, Hercules, CA, USA).

### 2.7. Immunoassay Kits

All immunoassay kits were measured in plasma. Myeloperoxidase (MPO) and xanthine oxidase (XOD) (Cusabio Technology LLC^®^, Houston, TX, USA), irisin (Cell Biolabs^®^, San Jose, CA, USA), and resolvin D1 (RvD1) (Cayman Chemical^®^, Ann Arbor, MI, USA) were determined using ELISA kits following the manufacturers’ instructions. Cytokeratin 18 (CK-18) concentration was determined using the M30 Apoptoense^®^ ELISA kit following the guidelines for use (PEVIVA^®^, in USA, Canada, and Japan). Tumor necrosis factor alpha (TNFα) and Interleukin-6 (IL-6) levels were determined in plasma using Human Custom ProcartaPlexTM (Invitrogen by Thermo Fisher Scientific, Bender MedSystems GmbH, Vienna, Austria) following the manufacturers’ instructions.

### 2.8. Statistics

Analyses were carried out with the Statistical Package for Social Sciences version 25.0 (SPSS Inc., Chicago, IL, USA). Descriptive statistics with mean ± SD (standard deviation) for participants’ baseline characteristics were used. Participants were classified in tertiles according to 6 months changed in IFC (T1: <−0.567, *n* = 20; T2: −0.567 to −7.13, *n* = 20; T3: >−7.13, *n* = 20). Differences among baseline characteristics according to tertiles of IFC were tested with one-way analysis of variance (ANOVA) and Bonferroni’s post hoc analysis when variables followed normal distribution or with Kruskal–Wallis test for non-normally distributed variables. Results were considered statistically significant if *p*-value < 0.05. Bivariate correlation between the difference of IFC levels and VO_2_ max were also analyzed with Pearson correlation.

## 3. Results

### 3.1. Anthropometric, Biochemical, and Hematological Parameters

The anthropometric and biochemical characteristics of participants with NAFLD categorized by tertiles after 6 months of change in IFC are shown in Table 1. Significant differences were evidenced in weight, BMI, triglycerides, alanine aminotransferase (ALT), and gamma glutamyl transferase (GGT) when comparing the evolution after 6 months of intervention. The patients in tertiles 2 and 3 presented greater decreases with respect to tertile 1 in weight (*p* = 0.017 and *p* < 0.001, respectively), BMI (*p* = 0.025 and *p* = 0.024, respectively), and triglycerides (*p* = 0.048 and *p* = 0.005, respectively), whereas patients in tertile 3 presented better ALT evolution with respect to tertile 1 (*p* = 0.014). No differences were observed in systolic and diastolic blood pressure or in the rest of the biochemical parameters.

Table 2 summarizes information on hematological parameters at baseline and 6 months follow-up according to tertiles of 6 months change in IFC. When changes between tertiles were compared, significant differences were reported in platelets levels, whereas no differences were described in the rest of the hematological variables of the participants.

**Figure 1:**
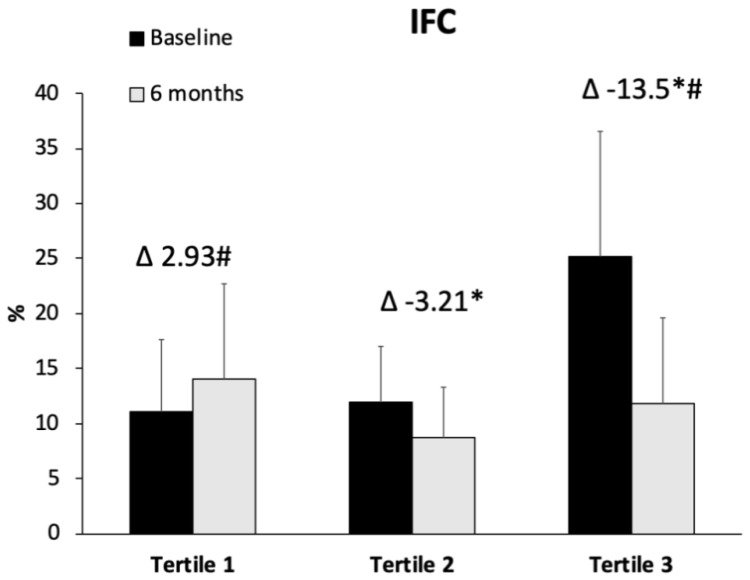
Values are the mean ± SD. Abbreviations: IFC, intrahepatic fat contents. Differences in means of IFC classified in tertiles was tested with one-factor ANOVA and Bonferroni’s post hoc analysis when variables followed a normal distribution or with Kruskal–Wallis test for non-normally distributed variables. * Differences with respect to tertile 1. ^#^ Differences with respect to tertile 2.

**Figure 2:**
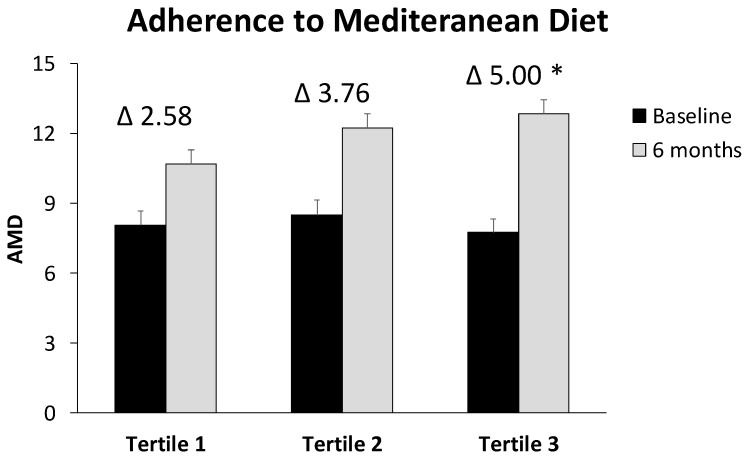
Values are the mean ± SD. Difference in means of ADM classified in tertiles was tested with one-factor ANOVA and Bonferroni’s post hoc analysis when variables followed a normal distribution or with Kruskal–Wallis test for non-normally distributed variables. * Differences with respect to tertile 1.

**Figure 3:**
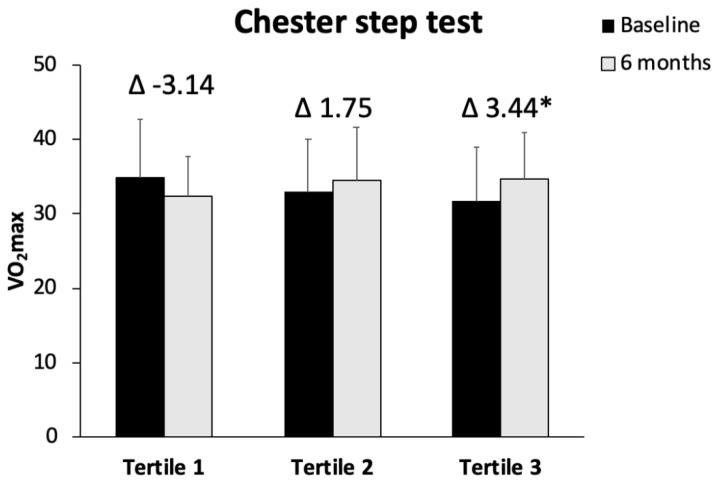
Values are the mean ± SD. Difference in means of IFC classified in tertiles was tested with one-factor ANOVA and Bonferroni’s post hoc analysis when variables followed a normal distribution or with Kruskal–Wallis test for non-normally distributed variables. * Differences with respect to tertile 1.

IFC levels at baseline and after 6 months of intervention were shown in Figure 1. The improvement in IFC was significantly higher in tertile 3 with respect to tertiles 2 and 1 (*p* < 0.001). The ADM was increased in the three group after 6 months when compared with the beginning of the intervention, and this improvement was significantly higher in the tertile 3 with respect to tertile 1 (*p* = 0.039) (Figure 2). The results of VO_2_ max in CST are represented in Figure 3. The obtained data reported a significant improvement in tertile 3 with respect to tertile 1 (*p* = 0.011). A direct correlation between the difference of IFC levels and VO_2_ max was found (*r* = 0.386, *p* < 0.01), whereas an inverse correlation was observed between IFC and ADM (*r* = −0.332, *p* < 0.05).

### 3.2. Oxidative Stress and Inflammatory Biomarkers

Table 3 shows the changes in the enzymatic activities of CAT and SOD and the protein levels of myeloperoxidase (MPO), xanthine oxidase (XOD), resolvin D1, irisin, and cytokeratin 18 (CK-18) and the biomarkers of plasma damage malondialdehyde (MDA) and protein carbonyl derivates. The differences in CAT activity after 6 months of intervention were significantly higher in tertile 3 with respect to tertile 1 (*p* = 0.002), whereas no significant changes were observed in SOD activity. Irisin and CK-18 levels reported differences in tertile 3 with respect to tertile 1, with higher reductions between baseline and 6 months (*p* = 0.047 for irisin and *p* = 0.042 for CK-18). No differences were found in resolvin D1, MPO, XOD, IL-6, TNFα, and MDA and protein carbonyls between groups.

## 4. Discussion

The main findings of the current study are that a reduction in IFC regardless of the type of intervention followed is related to better oxidative and inflammatory state and with an improvement in aerobic capacity. The baseline situation of these patients at the beginning of the study in addition to NAFLD diagnosed by MRI showed biochemical parameters out of the described reference values [12]. The reduction of weight and BMI in tertile 3 subjects when compared with tertile 1 is associated with the improvement in IFC. Previous studies suggested that BMI or weight reduction is not essential for decreasing hepatic fat contents or to restore normal liver function [42]; however, current international guidelines stated that the primary goal of nutrition therapy in NAFLD is to reduce energy intake by 500–100 kcal per day to achieve a 7–10% reduction in body weight [30,43,44]. It has been reported that an optimal nutritional therapy for patients with NAFLD could be an initial very-low-calorie diet period of several weeks, characterized by high protein, high-soluble fiber, and low-carbohydrate formula, followed by a structured program of food reintroduction implementing Mediterranean dietary patterns [45].

The intervention groups in the present study were designed to improve the IFC in NAFLD patients. For this reason, three different interventions for the management of NAFLD were selected following international guidelines, which recommend the combination of dietary modifications and physical activity to lose weight [46,47]. At 6 months of intervention, a similar improvement was observed in the three groups, with no statistical differences between them [47]. These results led us to analyze the differences in stress and inflammation markers according to the degree of improvement in the IFC regardless of the intervention followed by the patients. However, when grouping the participants according to the degree of improvement in IFC, it was observed that the groups are different at baseline. In fact, the group that presented the highest IFC at the beginning of the study is the one that improved the most after 6 months of intervention. These data are consistent with studies that report greater weight loss in patients undergoing nutritional intervention as their BMI increases due to the greater ease of losing fat in subjects with a higher initial fat content than in those closer to normal weight [48,49]. In addition, when analyzing the ADM, a greater increase was observed between the start of the intervention and after 6 months in parallel to the greater decrease in IFC. The Mediterranean diet is characterized by its antioxidant and anti-inflammatory properties, so the improvement in diet quality may contribute to the improvement observed in the patients included in tertile 3 [50].

Participants with better improvement in IFC showed fewer levels of triglycerides, ALT, and GGT after 6 months of intervention with respect to basal levels. One of the hallmarks of NAFLD is the appearance of insulin resistance that leads to an increase in plasma glucose concentrations and an excessive production of triglyceride rich VLDL, inducing hypertriglyceridemia [51,52]. ALT is a liver enzyme commonly used as specific marker of liver inflammation and hepatocellular damage and widely used as marker of NAFLD presence in addition to presenting a good correlation with liver fat contents [53,54]. However, it should be considered that up to 25% of patients with NAFLD had normal ALT values; therefore, high ALT serum levels might underestimate the prevalence of NAFLD [55]. The current findings are in accordance with previous results, which showed high levels of triglycerides, ALT, and GGT in NAFLD patients with IFC ≥ 2 in comparison to patients with IFC of 0 and 1 [12]. This improvement was also observed in a dietary intervention study based on the Mediterranean diet, in which the patients reduced their weight by 7% along with a significant improvement in BMI, waist circumference, AST, ALT, GGT, and TG after the intervention period [55].

One of the relevant results of the current study is the direct relationship between the improvement in aerobic capacity (VO_2_ max) and the improvement in IFC, indicating the importance of physical activity in the reversal of fat accumulation in the liver. It has been evidenced that the CST is a valid, easy, and inexpensive solution for assessing the VO_2_ max in individuals with hypertension [56]. Increasing physical activity (aerobic physical activity and resistance training) and avoiding a sedentary lifestyle has been shown to exert a beneficial impact on NAFLD by improving liver injury, liver fat, and the histologic features of NAFLD [57]. In this sense, it has been shown that a lifestyle intervention combining diet and physical activity promotion improved functional fitness in patients with NAFLD [58]. However, it has been shown that although patients with NAFLD are aware of the need for physical activity, most described problems for its regular practice, such as lack of resources and education, physical discomfort during exercise, or time constraints [59]. All make it necessary to motivate and work hard with patients with NAFLD so that they perform physical exercise and do not abandon it.

Moreover, current participants also showed a reduction of the activities of enzymatic antioxidants CAT and SOD after 6 months of intervention in tertile 3 subjects. Previous data suggested that the excessive ROS production and inflammation are key actors in the pathogenesis of NALFD and directly related to hepatic cell death and apoptosis [28]. These findings would indicate a reduction in the degree of oxidative stress and points out the importance of the improvement of IFC as a healthy life contributing for a better oxidative stress status. It has been well-established that the activities of enzymatic antioxidants were high in early and advanced NAFLD subjects compared to controls because of a more pro-oxidant state in these patients [12,60]. The alterations in lipid metabolism led to the accumulation of lipids in the liver, which results in an overproduction of ROS within the mitochondria electron transport chain. Moreover, metabolic liver diseases such as NAFLD are associated with increased ROS production during fatty acid β-oxidation, endoplasmic reticulum stress, and NADPH oxidase alterations [61]. The reduction in the activity of antioxidant enzymes is not related to changes in oxidative damage markers, which could indicate that the activation of antioxidant defenses in these patients allows keeping oxidative damage under control.

In addition, the current findings did not show significant differences in the MPO and XOD levels as biomarkers of pro-oxidative states. In the case of MPO, as this enzyme is released mainly by neutrophils, this lack of changes could be related to the fact that the number of these cells did not change after 6 months of intervention [62]. Regarding XOD, there is a trend to decrease, but this change is not significant; therefore, it would be necessary to achieve an additional improvement in hepatic steatosis with a longer intervention time.

Furthermore, irisin levels were significantly reduced in tertile 3 after 6 months with respect to tertile 1. Irisin is a myokine/adipokine induced by exercise in humans, which is proposed to produce “browning” of white adipose tissue, thus increasing thermogenesis and energy expenditure [63]. Irisin is a promising regulator of glucose metabolism, which is involved in glucose homeostasis in muscle, liver, and adipose tissue, contributing to normoglycemia [64]. Previous report showed that the subjects with NAFLD had higher irisin levels than healthy ones [65], and these high levels have been suggested to act as a compensatory mechanism aimed at improving energy metabolism and insulin sensitivity [66]. Thus, the reduction of its levels in the group with more improvement could be indicative of a metabolic normalization of these patients.

The current study revealed that patients with NAFLD, who improved the IFC levels the most, also showed an improvement in CK18 levels. CK18 is a marker for apoptosis and inflammation, which found to be increased in NAFLD patients [67]. Previous studies revealed that CK18 increased with liver steatosis, and it has been described as an adequate and non-invasive marker, which could allow for the identifications of patients with NAFLD [12,68,69]. Moreover, studies have shown a decrease in serum CK18 levels and weight loss in patients with liver fibrosis after 6-month dietary intervention; consequently, this points out the existence of a positive association between changes in CK18 levels and weight loss [70]. However, other more general markers related to inflammation, such as TNFα, IL-6, or resolvin D1, did not show changes in their values. This could be due to the fact that although after 6 months of intervention, there was a reduction in BMI, patients continue to be obese; thus, these more general markers would not be sensitive enough and would require further improvement.

A limitation of the study could derive from the differences in the baseline IFC between the groups, which could make it difficult to interpret the results. However, the objective of the study was to analyze the changes associated with the IFC after a 6-month period to evidence the benefits of liver fat loss rather than comparing the initial values.

## 5. Conclusions

The present data evidenced that the reduction of IFC is associated with an improvement in oxidative stress and pro-inflammatory status and a better cardiorespiratory fitness in NAFLD patients. Plasma oxidative stress and inflammation biomarkers were mainly reduced in the patients in the tertile that showed a greater reduction in intrahepatic fat levels after 6 months of lifestyle intervention. The relationship between aerobic capacity improvement, the ADM, and IFC reduction highlights the importance of the quality of diet and regular physical activity in the prevention/reversal of NAFLD. In conclusion, the beneficial effects of lifestyle changes on NAFLD development could be applied to prevent incident NAFLD (as cardiovascular diseases) and reduce the future public health burden. However, evidence regarding the effects of this approach on NASH with advanced fibrosis, until now, is insufficient and must be evaluated and verified in future studies.

## Figures and Tables

**Table 1:** Changes in anthropometric and biochemical characteristics of adults with nonalcoholic fatty liver disease (NAFLD) categorized by tertiles after 6 months of lifestyle intervention in intrahepatic fat content (IFC).

		Tertile 1 (<−0.567) *n* = 20	Tertile 2 (−0.567 to −7.13) *n* = 20	Tertile 3 (>−7.13) *n* = 20	*p*-Value
Weight (kg)	Baseline	91.5 ± 15.7	97.8 ± 16.2	93.9 ± 8.1	
	6 months	90.4 ± 14.9	92.7 ± 13.2	87.1 ± 8.53	
	Δ	−1.06 ± 3.36	−5.08 ± 4.83 *	−6.79 ± 5.06 *	<0.001
BMI (kg/m^2^)	Baseline	32.5 ± 3.30	34.4 ± 4.93	34.0 ± 2.70	
	6 months	32.2 ± 2.91	32.7 ± 4.48	31.5 ± 2.29	
	Δ	−0.375 ± 1.17	−1.71 ± 1.56 *	−2.51 ± 1.87 *	<0.001
Systolic blood pressure (mmHg)	Baseline	137.4 ± 20.5	134.0 ± 12.2	142.2 ± 16.9	
	6 months	134.0 ± 12.0	135.5 ± 11.1	132.7 ± 11.0	
	Δ	−3.74 ± 12.6	1.56 ± 13.1	−1.99 ± 30.4	0.318
Diastolic blood pressure (mmHg)	Baseline	80.8 ± 9.71	81.5 ± 7.03	84.3 ± 9.98	
	6 months	81.4 ± 7.55	82.0 ± 7.65	78.0 ± 9.05	
	Δ	0.031 ± 9.76	1.00 ± 6.83	−0.353 ± 18.4	0.155
Glucose (mg/dL)	Baseline	109.2 ± 23.5	116.1 ± 41.9	115.1 ± 20.4	
	6 months	110.1 ± 25.7	112.5 ± 43.7	111.7 ± 41.0	
	Δ	0.850 ± 17.9	−2.76 ± 11.0	−3.40 ± 27.7	0.253
Hb1Ac (%)	Baseline	6.09 ± 1.08	6.08 ± 1.13	6.00 ± 0.557	
	6 months	6.05 ± 0.886	5.85 ± 0.880	5.74 ± 0.412	
	Δ	−0.026 ± 0.456	−0.229 ± 0.384	−0.238 ± 0.447	0.235
Triglycerides (mg/dL)	Baseline	178.3 ± 81.8	163.0 ± 58.5	240.2 ± 140.6	
	6 months	232.2 ± 160.5	128.1 ± 34.2	159.4 ± 78.5	
	Δ	53.8 ± 159.7	−46.8 ± 34.1 *	−80.8 ± 145.5 *	0.002
HDL cholesterol (mg/dL)	Baseline	44.1 ± 11.9	42.2 ± 8.28	39.6 ± 6.94	
	6 months	44.7 ± 14.0	45.8 ± 10.9	39.9 ± 5.83	
	Δ	0.950 ± 6.59	4.24 ± 5.25	0.250 ± 4.51	0.054
LDL cholesterol (mg/dL)	Baseline	127.3 ± 34.5	125.2 ± 27.2	129.0 ± 28.8	
	6 months	128.5 ± 35.1	112.4 ± 25.8	121.6 ± 33.4	
	Δ	5.88 ± 33.8	−7.38 ± 25.0	−7.11 ± 21.3	0.266
Cholesterol total (mg/dL)	Baseline	224.0 ± 74.7	200.6 ± 32.1	214.8 ± 34.1	
	6 months	212.7 ± 38.5	183.6 ± 33.1	192.9 ± 39.2	
	Δ	−6.05 ± 78.9	−11.1 ± 26.1	−21.9 ± 35.5	0.616
Bilirubin (mg/dL)	Baseline	0.811 ± 0.527	0.650 ± 0.254	0.700 ± 0.395	
	6 months	0.739 ± 0.379	0.636 ± 0.267	0.820 ± 0.554	
	Δ	0.024 ± 0.371	0.003 ± 0.223	0.109 ± 0.300	0.516
AST (U/L)	Baseline	23.3 ± 8.33	22.8 ± 5.71	29.4 ± 10.6	
	6 months	23.9 ± 6.40	20.9 ± 5.52	23.9 ± 7.68	
	Δ	0.550 ± 6.42	−1.11 ± 7.83	−3.28 ± 7.12	0.246
ALT (U/L)	Baseline	27.9 ± 10.1	32.4 ± 19.1	60.8 ± 58.7	
	6 months	28.7 ± 11.9	24.0 ± 9.35	29.8 ± 13.0	
	Δ	−0.800 ± 8.46	−8.48 ± 15.4	−28.0 ± 50.8 *	0.004
GGT (U/L)	Baseline	44.2 ± 29.2	32.5 ± 14.3	48.8 ± 25.2	
	6 months	43.8 ± 23.8	27.8 ± 11.3	44.7 ± 55.5	
	Δ	−0.450 ± 19.5	−4.68 ± 5.71	−4.14 ± 40.4	0.022
CRP (mg/dL)	Baseline	0.498 ± 0.505	0.529 ± 0.509	0.529 ± 0.711	
	6 months	0.356 ± 0.394	0.435 ± 0.336	0.322 ± 0.272	
	Δ	−0.143 ± 0.494	−0.090 ± 0.318	−0.220 ± 0.749	0.750

Values are the mean ± SD. Abbreviations: BMI, body mass index; Hb1Ac, glycated hemoglobin 1A; HDL cholesterol, high-density lipoprotein; LDL cholesterol, low-density lipoprotein; AST, aspartate aminotransferase; ALT, alanine aminotransferase; GGT, gamma glutamyl transferase; CRP, c-reactive protein. Difference in means of IFC classified in tertiles was tested with one-factor ANOVA and Bonferroni’s post hoc analysis when variables followed a normal distribution or with Kruskal–Wallis test for non-normally distributed variables. * Difference with respect to tertile 1.

**Table 2:** Changes in hematological parameters of adults with nonalcoholic fatty liver disease (NAFLD) categorized by tertiles of 6 months change in intrahepatic fat content (IFC).

		Tertile 1 (<−0.567) *n* = 20	Tertile 2 (−0.567 to −7.13) *n* = 20	Tertile 3 (>−7.13) *n* = 20	*p*-Value
Hematocrit (%)	Baseline	43.7 ± 4.08	43.4 ± 4.11	44.9 ± 4.15	
	6 months	43.5 ± 3.45	44.3 ± 3.73	45.7 ± 2.76	
	Δ	−0.250 ± 2.00	0.629 ± 1.54	0.780 ± 2.58	0.244
Erythrocytes (10^6^/μL)	Baseline	4.90 ± 0.349	4.84 ± 0.465	5.07 ± 0.403	
	6 months	4.88 ± 0.329	4.89 ± 0.407	5.12 ± 0.289	
	Δ	−0.022 ± 0.216	0.040 ± 0.216	0.054 ± 0.342	0.217
Leukocytes (10^3^/μL)	Baseline	7.18 ± 2.16	7.68 ± 1.95	7.44 ± 1.50	
	6 months	6.92 ± 1.85	7.77 ± 1.96	6.94 ± 1.58	
	Δ	−0.253 ± 1.25	0.066 ± 1.60	−0.503 ± 0.691	0.533
Platelets (10^3^/μL)	Baseline	228.2 ± 49.4	239.1 ± 44.0	243.7 ± 53.3	
	6 months	226.9 ± 55.0	242.7 ± 46.7	226.2 ± 47.1	
	Δ	−1.30 ± 34.8	3.62 ± 23.7	−17.6 ± 27.4	0.022
Neutrophils (10^3^/μL)	Baseline	3.85 ± 1.45	4.37 ± 1.19	3.86 ± 0.953	
	6 months	3.66 ± 1.48	4.44 ± 1.63	3.76 ± 1.14	
	Δ	−0.189 ± 0.843	0.107 ± 1.30	−0.102 ± 0.616	0.914
Lymphocytes (10^3^/μL)	Baseline	2.35 ± 0.672	2.62 ± 0.805	2.67 ± 0.667	
	6 months	2.35 ± 0.516	2.47 ± 0.695	2.36 ± 0.671	
	Δ	0.002 ± 0.497	−0.151 ± 0.467	−0.315 ± 0.278	0.071
Monocytes (10^3^/μL)	Baseline	0.656 ± 0.266	0.599 ± 0.151	0.600 ± 0.101	
	6 months	0.613 ± 0.242	0.596 ± 0.159	0.566 ± 0.132	
	Δ	−0.043 ± 0.112	−0.003 ± 0.133	−0.034 ± 0.097	0.596
Eosinophils (10^3^/μL)	Baseline	0.266 ± 0.183	0.221 ± 0.148	0.244 ± 0.111	
	6 months	0.243 ± 0.139	0.207 ± 0.110	0.203 ± 0.129	
	Δ	−0.023 ± 0.113	−0.014 ± 0.067	−0.042 ± 0.094	0.310
Basophils (10^3^/μL)	Baseline	0.058 ± 0.026	0.058 ± 0.023	0.067 ± 0.025	
	6 months	0.059 ± 0.024	0.051 ± 0.024	0.054 ± 0.023	
	Δ	0.001 ± 0.026	−0.006 ± 0.029	−0.013 ± 0.033	0.325

Values are the mean ± SD. Difference in means of IFC classified in tertiles was tested with one-factor ANOVA and Bonferroni’s post hoc analysis when variables followed a normal distribution or with Kruskal–Wallis test for non-normally distributed variables.

**Table 3:** Oxidative stress and inflammatory biomarkers in the plasma of patients with NAFLD categorized by tertiles of 6 months change in IFC.

		Tertile 1 (<−0.567) *n* = 20	Tertile 2 (−0.567 to −7.13) *n* = 20	Tertile 3 (>−7.13) *n* = 20	*p*-Value
Enzymatic Activities					
CAT (K/L blood)	Baseline	41.2 ± 9.41	48.0 ± 12.3	61.8 ± 16.7	
	6 months	56.8 ± 29.0	37.2 ± 22.6	36.7 ± 22.9	
	Δ	13.9 ± 30.2	−7.84 ± 22.8	−21.9 ± 26.2 *	0.003
SOD (pkat/L blood)	Baseline	295.6 ± 60.9	283.4 ± 64.3	309.9 ± 75.6	
	6 months	311.9 ± 96.9	287.4 ± 65.7	287.8 ± 69.8	
	Δ	42.6 ± 156.3	9.47 ± 100.9	−24.9 ± 125.5	0.361
ELISA assays					
MPO (ng/mL)	Baseline	4.07 ± 2.89	4.99 ± 2.66	4.16 ± 2.22	
	6 months	3.26 ± 1.06	3.67 ± 1.69	3.14 ± 1.19	
	Δ	−0.874 ± 2.66	−1.15 ± 3.32	−1.04 ± 2.00	0.680
XOD (ng/mL)	Baseline	0.411 ± 0.122	0.370 ± 0.124	0.386 ± 0.087	
	6 months	0.460 ± 0.243	0.348 ± 0.161	0.350 ± 0.113	
	Δ	0.068 ± 0.261	−0.017 ± 0.166	−0.023 ± 0.106	0.307
Resolvin D1 (pg/mL)	Baseline	132.9 ± 44.2	135.1 ± 43.4	140.3 ± 33.8	
	6 months	147.5 ± 30.7	159.5 ± 45.5	162.6 ± 32.4	
	Δ	14.4 ± 36.4	24.0 ± 62.2	23.2 ± 25.8	0.706
Irisin (ng/mL)	Baseline	118.6 ± 76.3	102.4 ± 63.8	132.3 ± 72.7	
	6 months	124.7 ± 92.9	112.2 ± 70.9	92.1 ± 59.1	
	Δ	6.3 ± 76.2	11.6 ± 65.5	−39.5 ± 56.4 *	0.002
CK-18 (U/L)	Baseline	47.1 ± 24.6	71.1 ± 44.2	96.8 ± 55.4	
	6 months	42.4 ± 22.3	41.0 ± 20.7	54.5 ± 41.0	
	Δ	−1.04 ± 21.3	−29.6 ± 50.2	−44.2 ± 54.0 *	0.040
Multiplex Assay					
	Baseline	4.25 ± 0.217	4.13 ± 0.259	4.28 ± 0.520	
IL-6 (pg/mL)	6 months	4.34 ± 0.377	4.26 ± 0.424	4.34 ± 0.604	
	Δ	0.103 ± 0.342	−0.015 ± 0.117	0.035 ± 0.289	0.805
	Baseline	3.05 ± 0.543	3.85 ± 0.466	4.25 ± 1.74	
TNFα (pg/mL)	6 months	3.95 ± 0.441	3.79 ± 0.381	4.19 ± 1.76	
	Δ	−0.038 ± 0.399	−0.209 ± 0.284	−0.120 ± 0.338	0.355
Oxidative damage					
MDA (nM)	Baseline	1.88 ± 0.737	1.71 ± 0.640	2.01 ± 0.843	
	6 months	1.69 ± 1.41	1.43 ± 0.597	1.20 ± 0.483	
	Δ	−0.111 ± 1.71	−0.337 ± 1.01	−0.874 ± 1.09	0.244
Protein carbonyl (%)	Baseline	100.0 ± 67.7	123.2 ± 65.0	136.8 ± 74.2	
	6 months	95.9 ± 34.3	88.3 ± 31.3	84.7 ± 18.6	
	Δ	−4.1 ± 88.4	−35.5 ± 78.4	−50.9 ± 71.7	0.285

Values are the mean ± SD. Abbreviations: CAT, catalase; SOD, superoxide dismutase; MPO, myeloperoxidase; CK-18, cytokeratin 18; XOD, xanthine oxidase; MDA, malondialdehyde. Difference in means of IFC classified in tertiles was tested with one-factor ANOVA and Bonferroni’s post hoc analysis when variables followed a normal distribution or with Kruskal–Wallis test for non-normally distributed variables. * Difference with respect to tertile 1.

## Data Availability

There are restrictions on the availability of data for this trial, due to the signed consent agreements around data sharing, which only allow access to external researchers for studies following the project purposes. Researchers wishing to access the trial data used in this study can make a request to the corresponding author: pep.tur@uib.es.
